# Women in Neuroscience: Four Women’s Contributions to Science and Society

**DOI:** 10.3389/fnint.2021.810331

**Published:** 2022-01-26

**Authors:** Priscilla E. Yevoo, Arianna Maffei

**Affiliations:** ^1^Department of Neurobiology and Behavior, SUNY – Stony Brook, Stony Brook, NY, United States; ^2^Graduate Program in Neuroscience, SUNY – Stony Brook, Stony Brook, NY, United States

**Keywords:** women in neuroscience, molecular mechanisms, neuromodulation, addiction, neuroscience, diversity, equity

## Abstract

There has been increased cognizance of gender inequity and the importance of an inclusive and diverse approach to scientific research in recent years. However, the innovative work of women scientists is still undervalued based on reports of fewer women in leadership positions, limited citations of research spearheaded by women, reduced federal grant awards, and lack of recognition. Women have been involved in trailblazing work that paved the way for contemporary scientific inquiry. The strides made in current neuroscience include contributions from women who deserve more recognition. In this review, we discuss the work of four women whose groundbreaking scientific work has made ineffaceable marks in the neuroscience field. These women are pioneers of research and innovators and, in addition, contribute to positive change that bolsters the academic community and society. This article celebrates these women scientists, their substantial impacts in neuroscience, and the positive influence of their work on advancing society and culture.

## Introduction

Discourses about diversity, equity, and the representation of women have been at the forefront of social and institutional conversations worldwide (Clark and Hurd, 2020; Berryhill and Desrochers, 2021; Cortes, 2021). The year of 2020, accompanied by the COVID-19 pandemic and its restrictions, further highlighted racial and gendered inequalities (Cortes, 2021). The disparities that emerged in career stability for women during the pandemic emphasized the need to create policies in the social and work world to navigate diversity, equity, and inclusion fruitfully (Berryhill and Desrochers, 2021; Cortes, 2021; Machlovi et al., 2021). The scientific field must join in the societal shift toward evaluating cultures and practices to balance structural inequities. One of the ways to bolster these commitments to equity is by acknowledging brilliant women in the scientific arena with the recognition and accolades they deserve (Fairhall and Marder, 2020). Numerous women in neuroscience have made historic contributions to the field, furthering knowledge despite the challenges of gender discrimination and limited acknowledgment in the scientific community (Metitieri and Mele, 2020; Machlovi et al., 2021).

The contributions of countless women have been ignored or minimized, but their accomplishments have inspired younger female scientists (Metitieri and Mele, 2020). Thanks to increasing awareness and a significant cultural shift, there has been substantial advancement toward increasing representation and recognition of women in the field (Haak, 2002). An enormous amount of work needs to be done to level the playing ground in the neurosciences and sciences in general. As the cultural shift continues, women should be cited. Their intellectual contributions should be recognized, and equal opportunities for sharing these contributions with the scientific community and the public should be provided. Disseminating research findings is vital for advancing any field of scientific inquiry (Schrouff et al., 2019; Fulvio et al., 2021). Having the opportunity to see one’s science recognized in conferences and publications is essential for establishing a reputation and securing funding that provides continuity to one’s research.

Tremendous scientific progress has been made in neuroscience in the past few decades. Our comprehension of the molecular and cellular mechanisms that drive thoughts and behavior has been exponentially expounded. These advancements have been possible due to the theories and innovations of exceptional neuroscientists, many of whom are women. The scientific work of four of these remarkable women – Drs. Sandhya Koushika, Eve Marder, Mary Jeanne Kreek, Yasmine Hurd – are reviewed here. We choose to highlight the work of these women due to its impact on our scientific journeys and because their cutting-edge work has led to a better understanding of the nervous system and diseases that perturb it.

### Dr. Koushika: Novel Techniques for Studying Axonal Transport

Dr. Sandhya Koushika is a neuroscientist at the Tata Institute of Fundamental Research. Her research focuses on the molecular mechanisms that regulate axonal transport in *Caenorhabditis elegans* (*C. Elegans*). Axonal transport is a well-coordinated cellular process that involves the shuttling of substances, including organelles, by motor proteins along the axon. This delivery system is essential for neuronal function and survival (Mondal et al., 2011). Following her doctoral and postdoctoral studies in the United States, Dr. Koushika returned to her home country, India, to establish her laboratory (Sedwick, 2013).

Starting her laboratory allowed her to investigate questions she explored during her postdoctoral work. She was interested in retrograde axon transport and developing a genetic system that would track the exodus of endogenous proteins from the synapse. To investigate this, she needed an experimental setup for live imaging of retrograde transport in the worm. The existing methods at the time, including radiolabeled nerve growth factor (NGF), did not allow for dynamic tracking *in vivo* of specific proteins (Murthy et al., 2011). Dr. Koushika’s laboratory developed a tool to exclusively tag retrograde cargo and visualize its transport in neurons *in vivo* (Murthy et al., 2011). Their methodology is based on injecting Alexa Fluor 594-conjugated immunoglobulin G (IgG) anti-GFP antibodies into transgenic *C. Elegans* lines that stably express GFP tagged to the synaptic vesicle protein, synaptobrevin-1 (SNB-1), in neurons. Upon exocytosis, SNB-1:GFP can bind to the Alexa Flour 594 conjugate, allowing retrograde activity to be visualized in both red and green channels under the microscope upon endocytosis of the vesicle. The stability and intensity of the fluorescent antibody enable long-term imaging of retrograde transport of specific transmembrane proteins. Another challenge to studying axonal transport is that model organisms are usually anesthetized, typically slowing or suspending the process. In collaboration with Dr. Venkataraman, her laboratory set up a microfluidic approach to study cellular and sub-cellular events in individual worms. This approach allows for anesthetic-free immobilization of intact genetic model organisms in a membrane-based *polydimethylsiloxane* (PDMS) microfluidic device (Mondal et al., 2011). Utilizing this technique, her group began unraveling the regulation of the various steps involved in axonal transport, such as the fate of the motor protein that carries the cargo, and mitochondria transport (Kumar et al., 2010; Mondal et al., 2011, 2021). This innovative method allows for long-term imaging of events occurring over longer time scales than previously possible as organisms remain viable post-imaging. The setup is not limited to *C. Elegans* and can also be used for imaging Drosophila larvae and zebrafish larvae.

Supervising one of only two worm laboratories in India and the only one at her institute came with its challenges; however, Dr. Koushika has since assisted in expanding the worm community by training new laboratories on *C. Elegans* husbandry and other laboratory techniques (Sedwick, 2013). She is also a mentor dedicated to introducing students to research at a young age, as she did not know of any researchers growing up (Sedwick, 2013). She has provided opportunities for high school students to participate and familiarize themselves with the research environment to raise interest and awareness for scientific work. Dr. Koushika’s adventurous and creative character is unmistakable in her scientific work and her group’s artistic endeavors, bridging art and science to communicate their findings. She has a page on her website dedicated solely to laboratory art (Koushika Lab, 2016). Dr. Koushika’s work contributes to the advancement of basic research in India and worldwide, hence expanding the inclusivity scope of scientific studies and bridging the gap in research across countries.

### Dr. Marder: Neuromodulation and Dynamics of Small Neural Networks

Dr. Eve Marder is a University Professor and the Victor and Gwendolyn Beinfield Professor of Neuroscience at Brandeis University. At the start of her career, Dr. Marder questioned a common notion – connections in neural circuits were hard-wired to produce a specific and predictable pattern of output – well-accepted in the field (Nassim, 2018). Dr. Marder’s questions led to remarkable novel discoveries. Using the crustacean stomatogastric ganglion circuit (STG) as a model system, she discovered that neural circuits were plastic and could alter their activity in direct response to various neuromodulators. During her doctoral work Dr. Marder aimed to identify all the chemicals neurons used to communicate within the STG circuit. She discovered that the pyloric dilator (PD) neurons, influential in shaping the rhythmic motion of muscles controlling the pylorus, contained choline acetyltransferase activity but lateral pyloric (LP) neurons did not (Marder, 1976). PD used acetylcholine in its excitatory connections to the pyloric dilator muscles (Marder, 1974, 1976). Dr. Marder explored signaling by other neuromodulators, including dopamine, serotonin, and others, and how they interacted with neurotransmitters across synapses (Meyrand et al., 1992; Pulver et al., 2003). Her collaborative work with Dr. Eisen profoundly changed the trajectory of neuroscience research by revealing the flexibility of neuronal circuits. Using microelectrodes to record voltage and current, they investigated how the electrically coupled PD and anterior burster (AB) neurons modulate postsynaptic neurons and show that PD and AB neurons release different neurotransmitters that generate significantly different outputs (Marder and Eisen, 1984). Subsequent work revealed that these electrically coupled neurons differed in their sensitivity to neurotransmitters and neuromodulators, thereby accounting for the shifts in firing times of the pyloric pattern generator (Eisen and Marder, 1984). Their pioneering work on the STG heralded current knowledge about ion channel and neuromodulator diversity and function.

Dr. Marder’s breadth of knowledge and ideas are seemingly endless. Her laboratory is recognized as a leader in computational neuroscience. Dr. Marder’s group was among the pioneers who developed the dynamic clamp method, which models artificial electrical conductance that can be injected into target neurons to simulate ionic conductance and synaptic inputs allowing the study of theoretical circuits’ output (Sharp et al., 1993). Her group modeled negative feedback homeostatic mechanisms to dissect how neuronal function maintains stability despite perturbation. In collaboration with Dr. Abbot, Dr. Marder’s team computationally simulated multicompartment neurons and investigated how the spatial distribution of calcium conductance may alter the physiology of the neuron (Siegel et al., 1994). They observed that local calcium concentration regulates channel density and can produce realistic spatial distributions of channels which shaped realistic non-uniform current distributions that were electrical activity and cell morphology dependent (Sharp et al., 1993; Siegel et al., 1994). They concluded that this activity-dependent modulation is vital to compensate for long-term potentiation and depression destabilizing effects and that intrinsic and synaptic modification creates a plastic yet stable neural network system (Siegel et al., 1994). The biological relevance of this experiment was validated in cultured STG neurons in collaboration with Dr. Turrigiano and Dr. Abbott, where they demonstrated that STG neurons transition from regular tonic firing to bursting activity when isolated in culture (Turrigiano et al., 1994). The tonic firing could be reinstated by hyperpolarizing rhythmic stimulation that mediates an increase in intracellular calcium concentration, indicating that calcium conductance is implicated in maintaining homeostatic and flexible neural systems (Turrigiano et al., 1994).

The general principles from Dr. Marder’s work are applicable to neural networks of other organisms, including humans. Her studies open technically and conceptually novel avenues of investigation. Her findings reveal that neural activity necessitates intricate organization and constant fine-tuning, dependent on activity patterns through combinations of channels and receptors, to generate varying outputs and behaviors (Prinz et al., 2004; Bucher et al., 2005; Schulz et al., 2006). The breadth of Dr. Marder’s work relies on synergistic use of experimental and theoretical techniques, including electrophysiological, biophysical, computational, anatomical, biochemical, and molecular techniques.

In addition to her scientific achievements, Dr. Marder is well known for her stellar mentorship, fostering a host of trainees who went on to develop successful careers within and outside of academia (Yale University, 2011). Her innovative thinking is renowned in the field, as so many people have been influenced by Dr. Marder’s conceptual advances and inspired by her passion for science (Yale University, 2011). Her desire for training future generations of exceptional scientists led her to help establish one of the first undergraduate neuroscience programs in the United States in 1990 (University of Oregon, 2020). In the book “Lessons from the Lobster”, a biography of Dr. Marder’s life and scientific concepts, Charlotte Nassim describes Dr. Marder as a “supremely gifted scientist”, and she hits the nail right on the head (Nassim, 2018).

### Dr. Kreek: Innovative Treatment for Drug Addiction

Dr. Mary Jeanne Kreek was a physician-scientist who studied drug abuse and addiction. She was a Professor and Head of the Laboratory of the Biology of Addictive Diseases at The Rockefeller University, and Senior Physician of The Rockefeller University Hospital. Very early in her career, Dr. Kreek successfully developed methadone maintenance therapy to treat heroin addiction (Dole et al., 1966). In the 1960s, there was an opioid crisis due to a surge in heroin abuse; hence Dr. Kreek and her colleagues, Drs. Dole and Nyswander, set out to understand heroin addiction and investigate pharmacological approaches to treatment. A breakthrough contribution of Dr. Kreek’s work was identifying heroin addiction as a neurological disease with behavioral consequences (Stimmel and Kreek, 2000). This recognition was a significant shift in ideology in a world where addiction was primarily considered criminal behavior (Chandler et al., 2009).

A seminal work from the efforts of Dr. Kreek and her collaborators showed that a daily oral dose of methadone blocked the euphoric “high” and prevented withdrawal symptoms – general malaise, nausea, tremors (Dole et al., 1966). In this double-blind study, heroin addicts were provided methadone maintenance treatment weeks before intravenous injections of various narcotics, including heroin, and saline. The euphoric effects of heroin and other narcotic drugs were scored and found to be markedly attenuated by the prior administration of methadone (Dole et al., 1966). As a well-rounded scientist, Dr. Kreek initiated longitudinal studies of the physiological effects and safety of long-term methadone use, which led to the development of the earliest laboratory techniques (isotope dilution and gas chromatography) for measuring methadone and other opioids in blood and tissues (Kreek et al., 1972; Dole and Kreek, 1973; Kreek, 1973). This work propelled the U.S. Food and Drug Administration’s authorization of methadone maintenance for opiate addiction (Volkow and Koob, 2021).

Dr. Kreek further investigated how drugs of abuse alter gene expression in brain regions, including the nucleus accumbens and ventral tegmental area, and examined how chronic use of such drugs affected behavior (Leri et al., 2006; Yuferov et al., 2010). Utilizing animal models, her laboratory developed administration and behavioral paradigms reflective of drug addicts’ use. This approach was instrumental in identifying genes and biological pathways that confer a predisposition to addictive tendencies. Dr. Kreek and collaborators observed that mu-opioid receptor (MOR) mRNA levels in both the nucleus accumbens and frontal cortex significantly increased in rats exposed to cocaine during place preference (CPP) conditioning – a behavioral paradigm used to study pleasurable and aversive effects of drugs (Leri et al., 2006). However, the upregulation of MOR mRNA levels in the nucleus accumbens was reduced by methadone in a dose-dependent manner (Kreek, 1996; Leri et al., 2006). Her laboratory identified multiple genetic traits, including the first proof of a *cis*-acting polymorphism, a functional haplotype in the *PDYN* gene and substantially higher DNA methylation rate of the *OPRM1* gene in the lymphocytes of heroin addicts, associated with addiction (Stimmel and Kreek, 2000; Yuferov et al., 2010).

Beyond her scientific accomplishments, Dr. Kreek has been described as “an extraordinary role model and mentor, especially for women scientists”, which is evident in her trainees’ success and in the number of women drawn to science by reading about her accomplishments (Volkow and Koob, 2021). She cared about her patients and refuted the societal stigma against addicts. Methadone maintenance treatment remains a very effective treatment for opiate addiction today (Joudrey et al., 2021). Dr. Kreek recently passed away. The field of addiction neuroscience lost a true intellectual giant, but her work continues to inspire driven and exceptional scientists.

### Dr. Hurd: Epigenetic and Cellular Mechanisms Underlying Addiction

Dr. Yasmin Hurd is the Chair of Translational Neuroscience and the Director of the Addiction Institute at Icahn School of Medicine at Mount Sinai. Dr. Hurd’s research is grounded in a bidirectional translational research perspective that relies on animal models and human subjects. She employs multidisciplinary approaches to dissect the complex neurobiological systems and mechanisms underlying addiction. Recently, Dr. Hurd’s cannabis research has been at the forefront of conversations regarding the legalization of marijuana (Scherer and Barcott, 2015; Caulkins and Kilmer, 2016; Gupta, 2018; Stenson, 2021). Previously, cannabis was classified as an illicit drug due to its addictive properties making it illegal to possess it (Smart and Pacula, 2019). It has been the most abused illicit drug, and Dr. Hurd’s research has expanded the limited knowledge on the consequences of cannabis exposure on the human brain (Center for Disease Control and Prevention, 2017). She is a pioneer in work on the transgenerational effect of cannabis on the developing brain (Wang et al., 2004; Szutorisz et al., 2016). Dr. Hurd’s investigations indicate that marijuana use during pregnancy could lead to behavioral and cognitive impairment in the offspring (Wang et al., 2004). This research was conducted using *in situ* hybridization histochemistry to visualize mRNA expression of cannabinoid receptor type 1 (CB1) and major dopamine receptor subtypes, D1 and D2, in postmortem human fetal brains from mothers with and without documented evidence of cannabis use during pregnancy (Wang et al., 2004). Dr. Hurd and collaborators observed reduced D2 mRNA expression levels in the amygdala of human fetuses. The reduction was positively correlated with the consumption of marijuana during pregnancy (Wang et al., 2004). These results were groundbreaking because they demonstrated that marijuana does not only affect brain activity in users but can interfere with the development of specific neural circuits in a transgenerational manner.

Dr. Hurd’s extensive research on cannabis has highlighted the ying-yang feature of the compound. While most research has focused on tetrahydrocannabinol (THC), the psychoactive component of cannabis, and its adverse effects, Dr. Hurd’s laboratory has contributed substantial knowledge on cannabidiol (CBD), a non-psychotomimetic component of cannabis with antipsychotic and anxiolytic properties, to the field. Using a rat drug-seeking and self-administration behavior model, CBD attenuated heroin-seeking behavior reestablished by exposure to a conditioned stimulus and a normalization of the glutamatergic receptor (AMPA GluR1) and CB1 expression, previously disrupted in stimulus cue-induced heroin seeking, in the nucleus accumbens (Ren et al., 2009). A translational clinical study of this effect was conducted in a double-blind, randomized placebo-controlled trial that assessed the effects of CBD administration on drug cue-induced craving and anxiety in drug-abstinent heroin addicts. They observed significantly reduced craving and anxiety induced by the presentation of salient drug cues than neutral cues following CBD administration, corroborating their animal work (Hurd et al., 2019). Her innovative cannabidiol studies are essential to developing potential new treatments for opioid addiction. The push to legalize marijuana has placed her in the spotlight, as evident in her multiple media appearances, including podcasts, PBS and Netflix shows, and CNN documentaries.

Dr. Hurd’s outstanding contribution to society does not end in the laboratory, as she utilizes multiple avenues to communicate her research and engage with the public (Scherer and Barcott, 2015; Gupta, 2018; Stenson, 2021). Besides that, she is an avid advocate for diversity and equity, which is conspicuous in the diversity of her laboratory and her participation in programs that foster education and outreach to underserved communities (Clark and Hurd, 2020; Mount Sinai Today, 2020). As one of the few Black women at higher tiers of academia, she shares the implicit and explicit bias she faces in the academy and discusses steps to resolve it in the Nature Human Behavior article “Addressing racism and disparities in biomedical science” (Clark and Hurd, 2020). Dr. Hurd’s work inspires many, and she is a role model to many women in the sciences (Mount Sinai Today, 2020).

## Concluding Reflections

Women continue to make meaningful contributions to the field of neuroscience, advancing our understanding of the nervous system and paving paths for modern scientific exploration. The women we highlighted here, Drs. Sandhya Koushika, Eve Marder, Mary Jeanne Kreek and Yasmine Hurd, pioneered new methodologies and proposed conceptual advances that have made indelible marks on the field. Their research in worms, crustaceans, rodents and human subjects has laid a vital foundation for contemporary brain research. These women, and countless others, are helping change the narrative of science being considered a “male-dominated field” (Aguinis et al., 2018). Their stories and scientific contributions should continue to trend in all cultural spheres to reach aspiring young minds.

It has been repeatedly reported that diversity enhances excellence and innovation due to the variety of thoughts and the breadth and scope of research questions that can arise from different lived experiences (Swartz et al., 2019). Although there has been an increased awareness of the importance of diversity, inclusivity, and equity in science research, the data still indicates that women in scientific fields face persistent challenges and biases as they strive to attain success and take on leadership roles (Schrouff et al., 2019; Swartz et al., 2019; Fulvio et al., 2021). These reported hurdles, in turn, discourage other women from pursuing academic scientific research despite contemporary societal and cultural acceptance. Recognition and visibility of women’s contributions in neuroscience are vital to their recruitment and retention (Fairhall and Marder, 2020; Fulvio et al., 2021). With this article, we bring attention to four outstanding women scientists, highlight their impact on neuroscience, and the positive influence of their work on increasing enthusiasm for science in young generations and on advancing society. Our goal is to awaken an interest in learning more about diverse perspectives and instill in the reader the curiosity to explore further the breadth of ideas that contributed to the advancement of the neuroscience field.

## Author Contributions

PY and AM contributed to this work. Both authors contributed to the article and approved the submitted version.

## Conflict of Interest

The authors declare that the research was conducted in the absence of any commercial or financial relationships that could be construed as a potential conflict of interest.

## Publisher’s Note

All claims expressed in this article are solely those of the authors and do not necessarily represent those of their affiliated organizations, or those of the publisher, the editors and the reviewers. Any product that may be evaluated in this article, or claim that may be made by its manufacturer, is not guaranteed or endorsed by the publisher.
